# One-repetition submaximal protocol to measure knee extensor muscle strength among older adults with and without sarcopenia: a validation study

**DOI:** 10.1186/s13102-020-00178-9

**Published:** 2020-05-06

**Authors:** Pedro Pugliesi Abdalla, Andesron dos Santos Carvalho, André Pereira dos Santos, Ana Claudia Rossini Venturini, Thiago Cândido Alves, Jorge Mota, Dalmo Roberto Lopes Machado

**Affiliations:** 1 College of Nursing of the University of Sao Paulo at Ribeirao Preto (EERP/USP), Avenida dos Bandeirantes, 3900, Ribeirão Preto, SP 14040-902 Brazil; 2 Paulista University, Physical Education, Avenida Presidente Juscelino Kubitschek de Oliveira, w/ no, São José do Rio Preto, 15092-415 SP Brazil; 3 Center for Research in Physical Activity, Health and Leisure (CIAFEL), University of Porto, Rua Dr. Plácido Costa, 91, Porto, 4200-450 Portugal; 4 School of Physical Education and Sport of Ribeirao Preto at the University of Sao Paulo (EEFERP/USP), Avenida dos Bandeirantes, 3900, Ribeirão Preto, 14040-900 SP Brazil

**Keywords:** Anthropometry, Body composition, Muscle mass, Frailty, DXA, Aged, Frail Elderly, Knee Joint, Lower Extremity, Quadriceps Muscle

## Abstract

**Background:**

Dynamic knee extensor muscle strength is a valid measure among healthy older adults but has not been tested in the sarcopenia condition. This study’s objective was to test the validity of a one-repetition submaximal strength protocol to measure dynamic knee extension strength in older adults with and without sarcopenia.

**Methods:**

Ninety-four physically independent older adults (female: *n* = 64, 60 to 85 years; male: *n* = 29, 60 to 85 years) participated in this study in Brazil during 2016–2017. Sarcopenia was classified and isokinetic unilateral knee extension strength was measured at 60°/s. Bilateral dynamic knee extension strength was estimated with an extensor chair using one-repetition submaximal protocol. Validity was determined using Spearman’s correlation with isokinetic muscle strength.

**Results:**

The frequency of sarcopenia was 11.7%. Sarcopenic individuals presented lower body mass, body mass index and skeletal muscle index. Only chronological age was higher among the sarcopenic individuals. A high correlation was found between isokinetic unilateral knee extension strength and bilateral estimated one-repetition with submaximal protocol (r = 0.74; *p* <  0.001), when the presence (r = 0.71; *p* = 0.014) and absence of sarcopenia (r = 0.74; p <  0.001) were considered. The validity of the one-repetition submaximal protocol for bilateral knee extension was confirmed.

**Conclusions:**

The estimated measure of bilateral knee extension muscle strength can be used to monitor adaptations promoted by physical exercise for older adults with and without sarcopenia. The validation enable studies that will propose cutoff points to identify sarcopenia with this submaximal protocol. This will enable early diagnosis and better management of sarcopenia, a disease with adverse impacts for older adults.

## Background

Sarcopenia is a progressive and generalised skeletal muscle disorder, which is characterized by loss of muscle strength and muscle mass [1]. Was recognized as a disease [2] and listed in the International Classification of Diseases (ICD) with code M62.84 in September 2016. Its prevalence in the Brazilian older adults’ population is at 17% [3], while it is at 10% in the world [4]. Sarcopenia negatively affects an individual’s health, leading to motor dependence and increased risk of falls and premature death [5].

The number of older adults in the world is increasing exponentially and by 2050 there will be 1.5 billion [6]. At this time, low- and middle-income countries will be responsible for housing 1.2 billion [6]. This raises concerns for populous countries like China, India, Indonesia and Brazil. Thus, actions are necessary to identify and prevent sarcopenia, a chronic public health problem with a considerable economic impact [5].

Outcomes resulting from sarcopenia can be prevented if the condition is diagnosed early [4]. International associations have established parameters for the diagnosis of sarcopenia, such as the European Working Group on Sarcopenia in Older People-EWGSOP [1, 7], European Society for Clinical Nutrition and Metabolism Special Interest Group on cachexia-anorexia in chronic wasting diseases-ESPEN [8], International Working Group on Sarcopenia-IWGS [9], Society of Sarcopenia, Cachexia and Wasting Disorders-SCWD [10], Foundation for the National Institutes of Health-FNIH [11] and Asian Working Group for Sarcopenia (AWGS) [12]. All these have established that decreased skeletal muscle tissue indicators, together with low physical performance (all six guidelines) or muscle strength tests (EWGSOP, AWGS and FNIH), are necessary to establishing a diagnosis.

In regard to muscle strength, the EWGSOP has selected cutoff points for handgrip strength in order to establish the diagnosis of sarcopenia [13–15]. Similarly, AWGS also selected parameters of this muscle strength [16, 17]. The FNIH also recommends handgrip strength, but proposes its own parameters to identify the disease.

The most effective interventions to prevent or recover from sarcopenia include resistance training and proper nutrition [18], even among nonagenarians [19]. Among frail older adults, the earlier sarcopenia is identified, the better the results of interventions in terms of body composition and functional performance variables, such as muscle strength [18, 20].

Handgrip strength, however, is not sensitive to adaptations promoted by resistance training among frail older adults [9] or those with sarcopenia [21–23]. Handgrip strength loses its relationship with the muscle strength of the lower limbs, which in turn is sensitive to changes promoted by resistance training [21–23]. Additionally, the muscle strength of the lower limbs is strongly associated with the functional capacity and mobility of older adults, when compared to handgrip strength [7, 24]. Therefore, handgrip strength is associated with the consequences of sarcopenia [25] and can be used for initial screening, but not for following-up interventions that include exercises.

With aging, muscle mass and strength decline more intensively in the lower limbs than in the upper limbs [26, 27]. Up to the age of 70 years old, a loss of strength, from 10 to 15%, is experienced in the lower limbs per decade [28, 29]. After this age, a steeper decline, from 25 to 40%, is expected per decade [28, 29]. The main morphological difference, when young individuals are compared to older ones, is the thickness of the quadriceps muscle, though the difference in hamstring muscles is almost imperceptible [30–32]. These morphological changes directly impact an individual’s functionality because these changes result in decreased performance of knee extensor muscle strength and decreased maintenance of knee flexor muscle strength [27]. Decreases from 55 to 76% of isometric knee extensor muscle strength have been reported [33].

Knee extensor muscle strength declines more rapidly with aging than does handgrip strength [24], which facilitates the early detection of sarcopenia. Additionally, knee extensor muscle strength is required by functional tasks, such as walking, rising from a chair, and climbing stairs [34]; proper strength prevents falls and maintains the bone health of the proximal femur [35].

There is a lack of cutoff points for knee extensor muscle strength to indicate sarcopenia [36]; only two studies have proposed such indicators [36, 37]. These studies, however, adopted isometric knee extensor muscle strength, which is different from isotonic/dynamic muscle contraction, traditionally used in resistance training implemented with sarcopenic individuals [21]. Isometric contraction underestimates knee extensor muscle strength measured by isokinetic dynamometry [36], which is considered a reference point for the measure of muscle strength [38]. Thus, the development of dynamic knee extensor muscle strength and its maintenance at appropriate levels show greater relevance for the prevention of functional decline [39].

The one-repetition maximum strength test (1RM), used to measure dynamic knee extension strength executed in an extensor chair, is valid among healthy individuals and presents high correlation (r = 0.75) with isokinetic dynamometer measures [40]. Nonetheless, the high load necessary for performing it may involve risks when assessing frail older adults [41]. Additionally, the 1RM has not been validated among individuals with sarcopenia. Thus, the objective is to test the validity of a one-repetition submaximal protocol for dynamic bilateral knee extensor muscle strength among older adults with and without sarcopenia. Findings from this study may contribute to advancing global health and epidemiological research of sarcopenia treatment.

## Methods

### Participants

A cross-sectional study design was carried out to evaluate older adults physically independent, that living in the community, in the city of Ribeirão Preto. The study was conducted from October 2016 to May 2017. A total of 100 older adults were recruited, that consented to participate. The study’s inclusion criteria were: walk independently, not present diseases that restrict mobility or muscle strength, unstable cardiovascular conditions, acute infections, tumors, back pain, prostheses or any conditions that limited taking the tests. The study’s exclusion criteria were: Individuals with a diagnosis of cancer or uncontrolled diseases, who presented sequelae of stroke, experienced a loss of more than three kilograms (kg) weight in the last 3 months, had dementia that restricts understanding and taking tests, who did not complete all the stages, or desired to withdraw from the study. One participant was considered non-eligible (had tumor in his quadriceps) and five excluded (two opted to not perform the muscle strength test, two did not obtain the minimum score required on the cognitive test and one had acute infections). The final sample included 94 older adults (29 men and 65 women) aged between 60 and 85 years.

### Procedures

Each participant was assessed in a laboratory in the morning period. Data collection took place in two sessions and the same evaluator took all the measures. A cognitive test was applied in the first session, together with an anthropometry protocol, body composition assessment; handgrip strength and usual walking speed were also verified. Isokinetic muscle strength and dynamic knee extension strength were measured in the second session. The interval between the two sections ranged from three to five days.

The cognition was assessed using the short version of the Mini-Mental State Examination (MMSE), which presents a maximum score of 19 points [42]; individuals who scored ≤12 were considered inapt.

Age is expressed in whole years. Body mass were measured using a digital scale in kg (precision of 0.1 kg) and height with stadiometer in meters (m) with precision of 0.01 m. These measures followed conventional standards [43].

Body components were estimated using Dual energy X-ray absorptiometry (DXA), scanner Hologic®, model QDR4500W; version 11.2, Bedford, MA. The system measures bone mineral content, fat, and lean soft tissue (LST). Appendicular lean soft tissue (ALST) was the sum of the upper and lower limbs’ LST. The image of the limbs was isolated from the trunk and head using standard cuts generated by the software following the procedures provided in the manufacturer’s manual.

Handgrip strength was measured (kg) using a Jamar® dynamometer, model 5030 J1, as recommended by the American Society of Hand Therapists [44]. The participants made three attempts with their dominant hands, with one-minute intervals; only the highest measure for each was recorded [45, 46].

The usual gait speed test was performed for four meters, monitored by photocells (FSpeed; FE Sistemas®, Brazil) arranged at the beginning and end of the course [14]. Sensors were positioned at the participants’ waist height, the signal of which was picked up by two pairs of receivers arranged on tripods. The signals were transmitted via Bluetooth to a tablet using FSpeed software (v1.0; FE Sistemas®, Brazil), which recorded the time in seconds (s) and speed in meters per second (m/s). The participants were instructed to walk with their usual gait speed, as if they were walking on the street, for example, going to a store [47]. They were positioned with both feet touching the starting line demarcated on the ground and, after a verbal command, they began to walk. The test was repeated and the average of two trials was used.

Criteria established by the EWGSOP [1] were used to diagnose sarcopenia: decreased levels of muscle strength and muscle mass. Physical performance is tested for severity of sarcopenia. Muscle strength was considered low when handgrip strength was below 30 kg for men and below 20 kg for women [14]. Skeletal muscle mass index (SMI) was the criterion used for muscle mass, where ALST is divided by squared height (kg/m^2^). SMI below 7.26 was considered low for men and below 5.45 kg/m^2^ was considered low for women [48]. Physical performance was considered poor when usual walking speed was ≤0.8 m/s [14].

The right lower limb was tested using a Biodex isokinetic dynamometer model System 4 Pro, in order to determine the unilateral knee extensor muscle strength reference point. The participants sat on chair and the backrest was adjusted to enable the back of their legs to touch the end of the seat. To avoid additional movements, the trunk, hip and leg being tested (right leg) were secured by straps. The chair was positioned so as to allow the lateral epicondyle of the knee to be aligned with the dynamometer rotation axis. The tibia’s distal end was fixed by Velcro positioned 0.1 m from the lateral malleolus. The participants were allowed to become familiar with the activity and were asked to perform 10 submaximal repetitions, with an angular speed of 60°/s, followed by a three-minute rest. Following, the unilateral knee extension test was performed with five maximum repetitions, and the peak torque value of the concentric contraction at 60°/s in Newton units per meter (Nm) was recorded. The participants were verbally encouraged without visual feedbacks. This protocol is safe for the patellofemoral joint, skeletal muscles and the cardiovascular system [49]. The angular speed used is appropriate to obtain the peak torque and is recommended for the older adults [49]. The variable obtained was peak torque (Nm) for the unilateral knee extension.

The 1RM was estimated according to a submaximal repetitions protocol [50]. The protocol to estimate the dynamic knee extensor muscle strength was performed bilaterally in an extensor chair (Lion Fitness® and model LFS), with cable, weight plate support and pulley. The participants were instructed to sit on the extensor chair and the backrest was positioned so as to allow the back of the leg to touch the end of the seat, enabling the participants to perform the test comfortably. There was a warm up with 10 repetitions in the initial load stage (one weight plate of 8.73 kg). The load was increased after 2 minutes (two plates of 8.73 kg or 17.46 kg) and another eight repetitions were performed. The test was initiated after 3 minutes. The initial test load was 45% of the body mass for women [51] and 64% for men, considering the proportion of muscle strength between sexes [52]. The objective was to perform 10 repetitions at most, a limit that allows for a more accurate estimate [53]. Therefore, depending on how fit the participant was, these initial fixed loads could be increased or decreased in order to approximate the optimum limit and establish an estimate. Three attempts with three-minute intervals were allowed. If it proved impossible to reach an estimate, the protocol was performed again after a minimum interval of 24 h [52]. The variable obtained using the one-repetition submaximal protocol was an estimation of 1RM for bilateral knee extension.

### Statistical analysis

Mean, standard deviation, minimum and maximum values considering a confidence interval of 95% were used to describe the sample. Comparisons between men and women and between sarcopenic and non-sarcopenic individuals were performed using Student’s t test for independent samples. Normality of data was checked using the Shapiro-Wilk test (S-W) (*n* ≤ 50) or the Kolmogorov-Smirnov test (K-S) (*n* > 50). Spearman’s correlation test was used to test the validity of the protocol to estimate the 1RM for dynamic knee extensor muscle strength, considering the isokinetic dynamometer peak torque as the reference point. Spearman’s was used because individuals without sarcopenia did not present normality for the 1RM estimate (K-S = 0.103; *p* = 0.028) and peak torque (K-S = 0.148; *p* <  0.001), while those with sarcopenia did not present normality for the peak torque (S-W = 0.854; *p* = 0.048). The protocol was considered valid, since the coefficient of correlation presented high (r ≥ 0.7 up to 0.89) or very high values (r ≥ 0.9). The analyses were performed using the SPSS version 20 (Chicago, IL) considering the classification of sarcopenia and level of significance at α = 0.05.

## Results

Table 1 presents the descriptive data (mean, standard deviation, minimum and maximum values, along with confidence interval) for anthropometric, health and body composition characteristics of the subjects included in this study. Table 2 presents the descriptive data for the variables of muscle strength and mobility.

**Table 1:** Descriptive and comparative data of anthropometric, health and body composition characteristics of older man and women (*n* = 94) from Ribeirão Preto, Brazil

Variables / Groups	***M***	***SD***	Min	Max	Range	95% CI		Normality test	
						***LL***	***UL***	Value	***p***
Mini-Mental State Examination									
♀ nSc (*n* = 59)	17.37	1.80	13.00	19.00	6.00	16.90	17.84	0.229^k-s^	< 0.001
♀ Sc (n = 6)	17.50	1.52	15.00	19.00	4.00	15.91	19.09	0.902^s-w^	0.389
♂ nSc (*n* = 24)	17.38	1.86	13.00	19.00	6.00	16.59	18.16	0.828^s-w^	0.001
♂ Sc (n = 5)	18.75	0.50	18.00	19.00	1.00	17.95	19.55	0.630^s-w^	0.001
Age (complete years)									
♀ nSc	69.10	5.83	60.00	85.00	25.00	67.58	70.62	0.098^k-s^	0.200
♀ Sc	75.83^*^	5.19	69.00	84.00	15.00	70.38	81.28	0.982^s-w^	0.960
♂ nSc	70.25	7.02	60.00	85.00	25.00	67.29	73.21	0.963^s-w^	0.506
♂ Sc	74.50	6.66	69.00	84.00	15.00	63.91	85.09	0.878^s-w^	0.331
Body mass (kg)									
♀ nSc	67.79^†^	11.20	45.60	103.55	57.95	64.87	70.71	0.110^k-s^	0.073
♀ Sc	58.12	12.54	39.35	73.40	34.05	44.96	71.27	0.931^s-w^	0.585
♂ nSc	74.74	13.26	46.45	109.85	63.40	69.14	80.34	0.949^s-w^	0.254
♂ Sc	61.43	15.74	43.00	75.50	32.50	36.38	86.47	0.884^s-w^	0.356
Height (m)									
♀ nSc	1.56^†^	0.06	1.46	1.74	0.28	1.55	1.58	0.079^k-s^	0.200
♀ Sc	1.55	0.08	1.45	1.65	0.20	1.46	1.63	0.930^s-w^	0.578
♂ nSc	1.69	0.08	1.58	1.88	0.30	1.65	1.72	0.945^s-w^	0.209
♂ Sc	1.62	0.08	1.51	1.68	0.18	1.50	1.74	0.821^s-w^	0.145
BMI (kg/m^2^)									
♀ nSc	27.75	4.37	20.81	39.95	19.14	26.61	28.89	0.105^k-s^	0.164
♀ Sc	24.03^*^	3.48	18.33	27.51	9.18	20.38	27.69	0.918^s-w^	0.489
♂ nSc	26.16	3.59	17.48	31.08	13.60	24.64	27.67	0.947^s-w^	0.230
♂ Sc	23.27	4.92	18.98	27.73	8.75	15.43	31.10	0.751^s-w^	0.040
Appendicular Lean Soft Tissue (kg)									
♀ nSc	14.80^†^	2.39	11.10	22.48	11.38	14.18	15.42	0.107^k-s^	0.090
♀ Sc	11.82^*¥^	1.86	9.21	14.61	5.40	9.87	13.77	0.980^s-w^	0.950
♂ nSc	21.63	4.04	14.49	32.48	17.98	19.92	23.33	0.954^s-w^	0.331
♂ Sc	16.78^†^	2.96	12.61	19.50	6.89	12.07	21.49	0.912^s-w^	0.494
Skeletal muscle index (kg/m^2^)									
♀ nSc	6.06^†^	0.92	4.19	8.67	4.48	5.82	6.30	0.096^k-s^	0.200
♀ Sc	4.91^*¥^	0.37	4.29	5.40	1.11	4.52	5.29	0.941^s-w^	0.666
♂ nSc	7.54	0.93	5.81	9.41	3.60	7.15	7.94	0.963^s-w^	0.493
♂ Sc	6.36^†^	0.73	5.57	7.25	1.68	5.20	7.52	0.986^s-w^	0.935
Fat mass (kg)									
♀ nSc	28.40^†^	7.15	13.79	47.93	34.14	26.53	30.26	0.120^k-s^	0.034
♀ Sc	24.85	7.85	13.56	32.76	19.20	16.62	33.09	0.897^s-w^	0.354
♂ nSc	21.78	6.73	7.65	33.61	25.97	18.94	24.62	0.979^s-w^	0.885
♂ Sc	18.08	9.18	9.99	28.46	18.47	3.47	32.69	0.862^s-w^	0.266

Note. *CI* confidence interval, *LL* lower limit, *UL* upper limit, *♀ nSc* non-sarcopenic women, *♀ Sc* sarcopenic women, *♂ nSc* non-sarcopenic men, *♂ Sc* sarcopenic men, *: *p* < 0.05 vs ♀ nSc; †: *p* < 0.05 vs ♂ nSc; ¥: *p* < 0.05 vs ♂ Sc, *k-s* Kolmogorov-Smirnov, *s-k*: Shapiro-Wilk, *BMI*: body mass index

**Table 2:** Descriptive and comparative data of skeletal muscle strength and mobility characteristics of older man and women (n = 94) from Ribeirão Preto, Brazil

Variables / Groups	***M***	***SD***	Min	Max	Range	95% CI		Normality test	
						***LL***	***UL***	Value	***p***
Estimation of 1RM for bilateral knee extension (kg)									
♀ nSc	41.89^†^	16.15	8.73	81.51	72.78	37.68	46.10	0.107^k-s^	0.088
♀ Sc	30.32	7.40	20.96	39.29	18.34	22.55	38.08	0.879^s-w^	0.263
♂ nSc	71.06	25.55	36.98	151.37	114.39	60.27	81.85	0.907^s-w^	0.031
♂ Sc	50.89^†^	17.65	28.58	65.04	36.47	22.81	78.97	0.864^s-w^	0.275
No. of repetitions									
♀ nSc	6.51	2.47	1.00	10.00	9.00	5.87	7.15	0.187^k-s^	< 0.001
♀ Sc	7.67	1.63	5.00	10.00	5.00	5.95	9.38	0.916^s-w^	0.480
♂ nSc	7.33	2.32	3.00	10.00	7.00	6.36	8.31	0.892^s-w^	0.015
♂ Sc	7.25	2.22	4.00	9.00	5.00	3.72	10.78	0.801^s-w^	0.103
Peak torque at 60°/s for unilateral knee extension (Nm)									
♀ nSc	73.66^†^	26.65	19.50	150.20	130.70	66.72	80.61	0.075^k-s^	0.200
♀ Sc	68.47	18.28	50.20	97.90	47.70	49.28	87.65	0.911^s-w^	0.441
♂ nSc	124.30	47.09	49.20	240.40	191.20	104.41	144.18	0.938^s-w^	0.148
♂ Sc	92.88	36.33	44.30	130.90	86.60	35.06	150.69	0.966^s-w^	0.818
Handgrip strength (kg)									
♀ nSc	24.47^†^	4.39	12.00	33.00	21.00	23.33	25.62	0.118^k-s^	0.040
♀ Sc	20.00^*¥^	3.16	17.00	20.00	9.00	16.68	23.32	0.829^s-w^	0.104
♂ nSc	38.21	8.40	18.00	56.00	38.00	34.66	41.75	0.975^s-w^	0.778
♂ Sc	27.25^†^	1.89	26.00	30.00	4.00	24.24	30.26	0.791^s-w^	0.086
Usual walking speed (m/s)									
♀ nSc	1.23	0.36	0.63	2.51	1.88	1.14	1.33	0.118^k-s^	0.039
♀ Sc	1.17	0.28	0.76	1.48	0.72	0.88	1.46	0.940^s-w^	0.656
♂ nSc	1.30	0.33	0.80	2.40	1.60	1.16	1.44	0.881^s-w^	0.009
♂ Sc	1.22	0.25	0.99	1.57	0.58	0.83	1.61	0.895^s-w^	0.409

Note. *CI* confidence interval, *LL* lower limit, *UL* upper limit, *♀ nSc* non-sarcopenic women, *♀ Sc* sarcopenic women, *♂ nSc* non-sarcopenic men, *♂ Sc* sarcopenic men; *: *p* < 0.05 vs ♀ nSc; †: *p* < 0.05 vs ♂ nSc; ¥: *p* < 0.05 vs ♂ Sc; k-s: Kolmogorov-Smirnov; s-k: Shapiro-Wilk, *1RM* one-repetition maximum

Sarcopenia presented a frequency of 11.7% (*n* = 11) in the sample (*n* = 94). Women (*n* = 65) presented a frequency of 9.2% (*n* = 6), while men (*n* = 29) presented a frequency of 17.2% (*n* = 5). Only 3.2% of the participants carried out the handgrip strength test with the left hemi body. Only 5.3% out of the total reported the left leg to be dominant.

Men presented higher body mass in comparison to women (*p* = 0.029), as well as height (*p* <  0.001), estimated 1RM for knee extension (*p* <  0.001), peak torque of knee extension (*p* <  0.001), handgrip strength (*p* < 0.001), ALST (*p* < 0.001) and SMI (*p* < 0.001), except for fat mass, which was higher among women (*p* < 0.001).

Sarcopenic individuals presented lower body mass (*p* = 0.029), BMI (*p* = 0.012) and SMI (*p* = 0.013), compared to their non-sarcopenic counterparts. Only chronological age was higher among the sarcopenic individuals (*p* = 0.002). Specific comparisons between sexes, together with the classification of sarcopenia, are presented in Tables 1 and 2, represented by *, † and ¥.

**Fig. 1:**
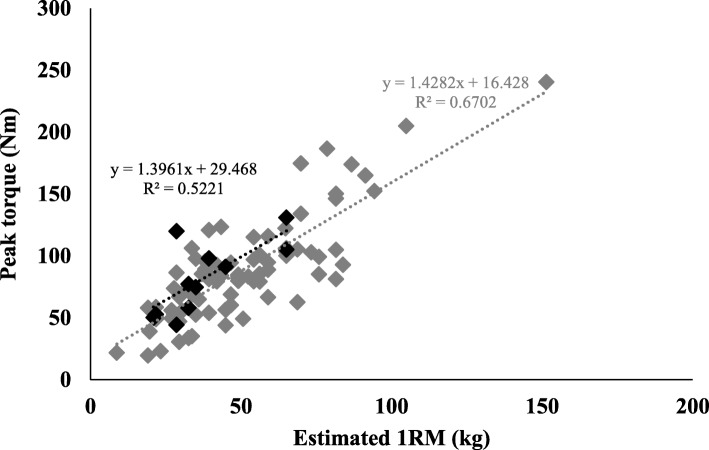
Dispersion between estimated 1RM and peak torque-60°/s for knee extension for sarcopenic and non-sarcopenic older adults. Legend: Black diamonds: sarcopenic older adults, Grey diamonds: non-sarcopenic older adults, 1RM: one-repetition maximum

To validate the estimated 1RM for knee extension, its correlation was tested with the reference measure (peak torque for knee extension). A high correlation was found between these when the entire sample was taken into account (r = 0.736; *p* < 0.001). The correlation was high when non-sarcopenic (r = 0.744; *p* < 0.001) and sarcopenic (r = 0.709; *p* = 0.014) older adults were analyzed separately. Thus, the validity of the estimated 1RM for knee extension was confirmed for the both participants, with and without disease. Dispersions between the two measures are presented in Fig. 1, which also presents the predictive linear equations originating from the trend lines between the two variables. Grey represents the equation for the participants without sarcopenia and black represents those with sarcopenia. The equation for the entire sample is “y = 19.353+1.397*x” (r^2^ = 0.65; SEE = 23.4 Nm).

## Discussion

The main goal of this study was to validate of a one-repetition submaximal strength protocol to measure dynamic knee extension strength in older adults with and without sarcopenia. The submaximal protocol was validated for both sarcopenic e non sarcopenic older adults. This proposal advances the field of sarcopenia studies by validate a clinical practice protocol that will contribute to the diagnosis of sarcopenia because it is easy to use, inexpensive and sensitive to the adaptations promoted by physical exercise. The validation will allow the creation of cutoff points to identify sarcopenia from the Dynamic knee extensor muscle strength. Especially in low- and middle-income countries these parameters will improve the understanding of the prevalence, incidence, risk factors, prevention and treatment of sarcopenia.

The dynamic knee extensor muscle strength decreases before handgrip strength does [54], which favors the early identification of sarcopenia. Such identification can support interventions that include exercises to recover from sarcopenia and ensure the muscle functionality of older adults [18, 20]. The dynamic knee extensor muscle strength one-repetition protocol had not yet been validated among older adults with sarcopenia. According to the literature, only one study [40] tested the validity of a 1RM protocol among healthy older adults. Nevertheless, its application includes heavy loads that compromise the safety of older adults with sarcopenia.

The method recommended to assess dynamic muscle strength among older adults in clinical practice is the 1RM [55]. Even though it involves a low risk of lesion (2.4%) among the older adults [56], age and some health conditions, such as sarcopenia/osteoporosis, may compromise the safety of utilizing it [41]. The development of a one-repetition submaximal protocol to estimate 1RM is justified because there is a concern over safety, since the original protocol adopts a very heavy load for one repetition. Such a load may impose exaggerated stress on muscles, bones or connective tissues, causing injuries because the stress exceeds the tensile resistance of these structural components [50]. The impact on bones is of concern, especially among older adults with osteoporosis and sarcopenia, because the risk of fracture among these individuals is 3.5 times greater than among healthy individuals or those with only one of these conditions [41]. Additionally, the 1RM trial raises blood pressure beyond the levels achieved when the submaximal protocol is used. Another concern is the highly specialized prerequisite of skill required by the 1RM protocol [50]. Such a concern is even more relevant when adolescent or older adults’ populations are tested.

Concurrent validity of the 1RM measure using the extensor chair and leg press was tested with 55 individuals with homogeneous characteristics (from 19 to 84 years old) [40]. They were divided between a young (< 60 years old) and an older adult (≥ 60 years old) group [40]. The isokinetic dynamometer was used as a reference, with the peak torque measure in Nm units of the isometric right knee extension at 80° and the isokinetic at various speeds (120°, 180°, 240° and 300°/s). Pearson’s correlation was used to validate the 1RM. When knee extension was performed at 120°/s speed (closest to present study) a high correlation was found among older adults (r = 0.75) for the 1RM obtained with extensor chair. For the 1RM made in leg press the correlation was only moderate (r = 0.60). The conclusion is that the 1RM obtained in the extensor chair is valid for assessing muscle strength among healthy older adults. The sample of older adults was small (*n* = 22) and was not divided between individuals with and without sarcopenia. The peak torque (92.0 ± 4.0 Nm) was higher than that found in present study for older adults with sarcopenia (75.4 ± 32.6 Nm) and also for those without sarcopenia (87.3 ± 39.8 Nm).

In the study of Verdijk [40] the validity of the knee extensor test was not obtained among older adults with sarcopenia. Additionally, the 1RM test was performed, which can be difficult for frail populations. Therefore, it was opted to validate the estimated 1RM protocol using an extensor chair [50], which is more easily applied with individuals who experience decreased motor capabilities, because it employs smaller loads than the maximum test. Additionally, in the present study, the older adults were classified in terms of sarcopenia and the sample was larger (*n* = 94). The coefficient of correlation was considered to be high (r > 0.70), both for the entire sample (r = 0.74), and for the groups with (r = 0.71) and without (r = 0.74) sarcopenia. This shows that even though the estimated 1RM for knee extension using the extensor chair obtained by the submaximal repetitions protocol is an indirect measure, it is valid to measure the muscle strength of lower limbs.

The prevalence of sarcopenia in Brazil has been reported in a meta-analysis/systematic review [3]. Thirty-one studies were selected, with 9416 individuals older than 60 years old, living in the community, in long-term care institutions or hospitalized. The prevalence of sarcopenia reported was 16%. In the present study, a frequency of 11.7% was found. Such a figure is lower than what is reported nationally, possibly because in the current study only physically independent older adults living in the community were recruited. The frequency would likely be higher if hospitalized individuals or those living in long-stay institutions were recruited. A larger number of frail individuals would be addressed and, consequently, the prevalence of sarcopenia would be higher. As submaximal strength protocol to measure dynamic knee extension strength is valid for older people with higher or lower muscle strength levels. The next step is to propose cutoff points based on this protocol to identify sarcopenia. In addition, lower limb muscle strength may facilitate early identification of the disease and measures of dynamic muscle strength are sensitive to exercise-induced adaptations in the older adults with sarcopenia.

Some limitations of the present study need to be highlighted. Other statistical indicators could be more appropriate to check the validity of estimated 1RM for knee extension, such as the Bland-Altman plot [57]. This plot verifies agreement between estimated and reference measures. However, the units between the two devices (isokinetic dynamometer [Nm] and extensor chair [kg]) are different, hindering the application of these resources. Thus, in this case, correlation is more appropriate to check for concurrent validity [40]. The study by Verdijk [40] and the current measured muscle strength using the isokinetic dynamometer only on the right lower limb. However, there are no differences between limbs in terms of peak torque among older adults using a 60°/s speed [58], which is the same angular velocity used in the present study. In the present study, the execution speed (cadence) was not controlled in the 1RM estimation protocol for knee extension. Even with the attempt to control the speed, it would be difficult to reproduce the angular speed of the isokinetic dynamometer. In addition, it is necessary to highlight that the 1RM estimation protocol showed a high correlation with the isokinetic dynamometer only at the angular speed of 60°/s. This does not allow generalizing the findings to other angular velocities. One factor that may have prevented the finding of an even higher correlation between the two measures is the bilateral strength deficit (BLD). BLD is characterized by reduced performance during synchronous bilateral limb contractions compared with the sum of identical unilateral limbs contractions [59]. In the present study, was measured muscle strength for knee extension unilateral (right leg isokinetic peak torque) and bilateral (1RM for knee extensor muscles), which can characterize the occurrence of BLD. The deficit is found in older adults [60] but primary cause of this remains equivocal [59]. However, it happens due to the inability of the neuromuscular system to generate maximal force during bilateral contraction [59]. Another limitation is noted by the heterogeneity of the studied sample, which makes it difficult to associate the results with age, sex and functional level. However, the sample heterogeneity allowed the recruitment of sarcopenic seniors, who are generally older, have less body mass, body mass index [61] and are more sedentary [62]. Despite the low number of sarcopenic older adults studied (six women and five men), the frequency of the disease obtained (11.7%) was similar to the global prevalence reported in the literature (10%) [4].

## Conclusion

The estimation of 1RM for bilateral knee extension obtained by submaximal repetition protocol for dynamic muscle strength was successfully validated among older adults with and without sarcopenia. Thus, the estimated measure can be used to monitor adaptations proposed by interventions involving exercise even for frail individuals. Cutoff points for dynamic and estimated knee extensor muscle strength need to be proposed to identify sarcopenia. This study will advance by providing health professionals an alternative with low cost to diagnose and monitor sarcopenia. Thus, contributing to reduce the adverse health effects of sarcopenia in the older adults.

## Data Availability

The datasets used and/or analysed during the current study are available from the corresponding author on reasonable request. Correspondence to: Pedro Pugliesi Abdalla, M.Sc., PT. Kinanthropometry and Human Performance Laboratory (LaCiDH). Av. Bandeirantes, 3900, ZIP Code: 14040–907. Ribeirão Preto - SP, Brazil. Tel: + 55 16 3315–0342. e-mail: pedroabdalla11@gmail.com

## Abbreviations

ICD: International Classification of Diseases
EWGSOP: European Working Group on Sarcopenia in Older People
ESPEN: European Society for Clinical Nutrition and Metabolism Special Interest Group on cachexia-anorexia in chronic wasting diseases
IWGS: International Working Group on Sarcopenia
SCWD: Society of Sarcopenia, Cachexia and Wasting Disorders
FNIH: Foundation for the National Institutes of Health
1RM: One-repetition maximum strength test
DXA: Dual energy X-ray absorptiometry
LST: Lean soft tissue
ALST: Appendicular lean soft tissue
S-W: Shapiro-Wilk test
K-S: Kolmogorov-Smirnov test
