# Correction to: One-repetition submaximal protocol to measure knee extensor muscle strength among older adults with and without sarcopenia: a validation study

**DOI:** 10.1186/s13102-020-00184-x

**Published:** 2020-05-26

**Authors:** Pedro Pugliesi Abdalla, Anderson dos Santos Carvalho, André Pereira dos Santos, Ana Claudia Rossini Venturini, Thiago Cândido Alves, Jorge Mota, Dalmo Roberto Lopes Machado

**Affiliations:** 1 College of Nursing of the University of Sao Paulo at Ribeirao Preto (EERP/ USP), Avenida dos Bandeirantes, 3900, Ribeirão Preto, SP 14040–902 Brazil; 2 Paulista University, Physical Education, Avenida Presidente Juscelino Kubitschek de Oliveira, w/ no, São José do Rio Preto, SP 15092–415 Brazil; 3 Center for Research in Physical Activity, Health and Leisure (CIAFEL), University of Porto, Rua Dr. Plácido Costa, 91, 4200–450 Porto, Portugal; 4 School of Physical Education and Sport of Ribeirao Preto at the University of Sao Paulo (EEFERP/USP), Avenida dos Bandeirantes, 3900, Ribeirão Preto, SP 14040–900 Brazil

**Correction to: BMC Sports Sci Med Rehabil (2020) 12:29**

**https://doi.org/10.1186/s13102-020-00178-9**

Following publication of the original article [1], the authors identified an error in the author name of Anderson dos Santos Carvalho.

The incorrect author name is: Andesron dos Santos Carvalho

The correct author name is: Anderson dos Santos Carvalho

The author group has been updated above and the original article [1] has been corrected.
